# Photoactivation of Cell-Free Expressed Archaerhodopsin-3 in a Model Cell Membrane

**DOI:** 10.3390/ijms222111981

**Published:** 2021-11-05

**Authors:** Navid Khangholi, Marc Finkler, Ralf Seemann, Albrecht Ott, Jean-Baptiste Fleury

**Affiliations:** Experimental Physics and Center for Biophysics, Saarland University, 66123 Saarbrücken, Germany; navid.khangholi@physik.uni-saarland.de (N.K.); marc.finkler@uni-saarland.de (M.F.); r.seemann@physik.uni-saarland.de (R.S.)

**Keywords:** Archaerhodopsin-3, lipid bilayer, microfluidics, cell-free gene expression

## Abstract

Transmembrane receptor proteins are located in the plasma membranes of biological cells where they exert important functions. Archaerhodopsin (Arch) proteins belong to a class of transmembrane receptor proteins called photoreceptors that react to light. Although the light sensitivity of proteins has been intensely investigated in recent decades, the electrophysiological properties of pore-forming Archaerhodopsin (Arch), as studied in vitro, have remained largely unknown. Here, we formed unsupported bilayers between two channels of a microfluidic chip which enabled the simultaneous optical and electrical assessment of the bilayer in real time. Using a cell-free expression system, we recombinantly produced a GFP (green fluorescent protein) labelled as a variant of Arch-3. The label enabled us to follow the synthesis of Arch-3 and its incorporation into the bilayer by fluorescence microscopy when excited by blue light. Applying a green laser for excitation, we studied the electrophysiological properties of Arch-3 in the bilayer. The current signal obtained during excitation revealed distinct steps upwards and downwards, which we interpreted as the opening or closing of Arch-3 pores. From these steps, we estimated the pore radius to be 0.3 nm. In the cell-free extract, proteins can be modified simply by changing the DNA. In the future, this will enable us to study the photoelectrical properties of modified transmembrane protein constructs with ease. Our work, thus, represents a first step in studying signaling cascades in conjunction with coupled receptor proteins.

## 1. Introduction

In biological cells, transmembrane proteins are embedded in the plasma membrane, composed of a lipid bilayer [1,2,3]. Certain transmembrane proteins act as receptors for external stimuli [4,5] such as light, mechanical stress, or the presence of specific molecular compounds [5,6,7,8,9]. Transmembrane receptor proteins transform the stimulus into signals that can be processed further downstream by the molecular machinery of the cell [2,4,10]. The stimulus produces structural or conformational changes in the transmembrane protein that will, for instance, result in an activation or deactivation of a specific ion channel, the release of a G protein (G protein-coupled receptors) or the activation of certain enzymes (enzyme-coupled receptors) [1,2,6,9,11,12,13].

**Figure 1:**
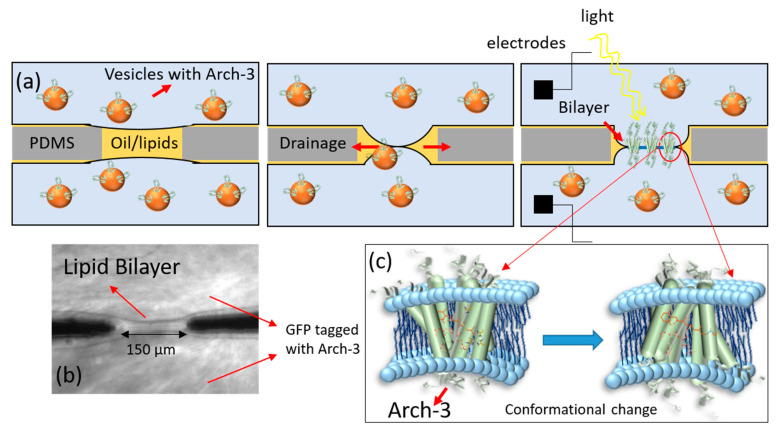
(**a**) A schematic of the formation of a lipid bilayer at the intersection of two microfluidic channels. First, the channel is filled with oil–lipid solution. Then, a cell-free expression reaction solution containing synthesized Arch-3-EGFP fused to the membrane of vesicles is injected into both channels. A remaining oil–filled lipid bilayer separates the two channels (left). Lipids from the oil phase and lipid vesicles with the inserted Arch-3-EGFP from the aqueous phase form a monolayer at both oil–water interfaces. As oil drains into the PDMS, the two interfaces gently meet to form a lipid bilayer containing Arch-3-EGFP (middle). The visualization of the inserted Arch-3-EGFP is achieved by exciting EGFP with blue light. AgCl electrodes inserted into the microfluidic device are used for electrical measurements (right). (**b**) The image shows the fluorescent signal of the EGFP tag from Arch-3 at the site of the suspended bilayer; PDMS elements remain dark. (**c**) The image shows the conformational changes of Arch-3 upon light exposure mediated by the deprotonation of retinal. This results in proton pumping which can be detected using electrophysiological measurements (scheme based on channel rhodopsin, adapted from [22]).

Archaerhodopsin proteins (Arch) are among a class of transmembrane receptor proteins called photoreceptors that react to light [14,15]. Upon illumination with green light, Arch will undergo deprotonation of a Schiff base, resulting in proton pumping [16,17,18,19]. In this way, Arch actively transports protons through the membrane and out of the cell [14,18,19]. In vivo, the resulting proton gradient enables ATP synthases to produce ATP [18,20]. Photoreceptors typically consist of seven transmembrane helices and the chromophore retinal [16,18,21]. The structure of Arch corresponds to G protein-coupled receptors that include rhodopsin. Because bacteriorhodopsin and Arch share similar structures and functions, their activation is assumed to be the same (see Figure 1c). Rhodopsin acts as a photoreceptor as well [1,15,22]. However, Arch acts as an ion channel, not as a G protein-coupled receptor. There are different kinds of Arch, which have slightly different properties [21]. Besides being photo active, Arch-3 fluorescence is also voltage sensitive [16,23], and it is often used in optogenetics as a voltage sensor [16,17]. To our knowledge, an electrophysiological characterization of Arch-3 has not yet been reported in the literature [18].

In this work, we present a new and simple microfluidic approach [24] to study the conducting properties of ion channels. We incorporated recombinantly produced Arch-3-EGFP into a free-standing lipid bilayer that mimics a biological membrane. A cell-free expression system was used for the production and reconstitution of Arch-3-EGFP. Such in vitro systems enable better control of various biochemical parameters and processes than in vivo systems [25,26,27,28]. They are regularly used to study gene circuits or reaction cascades [29,30,31]. In vitro, proteins, in the form of wild-type proteins or with modifications, can easily be produced recombinantly by the simple addition of the corresponding coding DNA [32,33,34]. Moreover, in vitro systems can mimic in vivo systems, allowing the transfer of results to the in vivo situation [35,36,37]. Using this approach, we report the first electrophysiological characterization of single Arch-3 channels in a model cell membrane. The method presented here (sketched in Figure 1) can be understood as a first step towards the further investigation of different signaling cascades.

## 2. Results and Discussion

**Figure 2:**
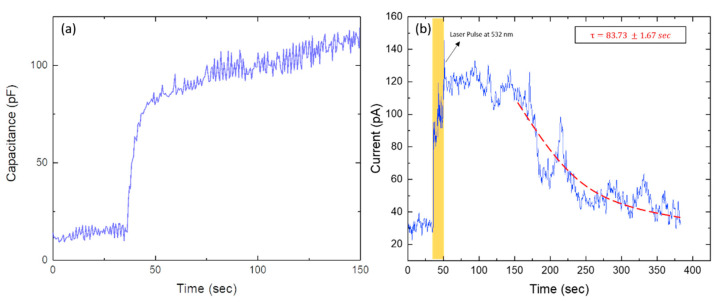
(**a**) Electrical capacitance as measured between the two microfluidic channels, separated by an oil phase (see Figure 1a) as a function of time. The signature of bilayer formation is the sudden increase in capacitance. (**b**) A real-time current recording of the bilayer containing Arch-3-EGFP. In the absence of light, we observe a current signal fluctuating around a constant value. The sudden jump in the current signal demonstrates the activation of Arch-3 caused by a green laser pulse applied to the bilayer at ~40 s ≤ t ≤ 50 s. The signal decays, with a time constant of ~84 s, to its initial dark value. The recovery time corresponds to the re-protonation of the Schiff base as reported in [38,39,40].

A DOPC/DPhPC bilayer containing Arch-3-EGFP was formed in a microfluidic device as described in the method section and as sketched in Figure 1. To verify the formation of a lipid bilayer, electrophysiological measurements were performed by applying a potential difference of 20 mV between both channels containing the reaction solution. The lipid bilayer separated the two ion-conducting water reservoirs, whereas the water–oil–water sandwich acted as a capacitor. Measuring the capacitance of such a sandwich in real time enabled the detection of bilayer formation; the graph in Figure 2a shows the related data from our experiments. The initial signal fluctuating around 10 pF corresponds to the situation with two monolayers separated by a macroscopic oil layer. The jump in the capacitance signal corresponds to the formation of a bilayer, a so-called zipping process. The following gradual increase in the capacitance demonstrates the growth of the bilayer area. This is due to the drainage of the oil to the PDMS at the plateau border.

Hydrostatic control of the flows enabled us to keep the bilayer area fairly constant for 1 h. The fluorescent image of EGFP tagged to Arch-3 under blue illumination, as shown in Figure 1b, confirmed the presence of Arch-3-EGFP in the vicinity of the lipid bilayer. After switching the laser illumination from blue to green, Arch-3 was activated, and an ion current was detected across the suspended lipid bilayer in real time upon light stimulation. The graph in Figure 2b shows the current intensity as a function of time, measured in the absence of light and after a short (~10 s) exposure to green laser light (532 nm) at ~40 s. Before light exposure, the current stayed almost constant. During the light pulse, the electrical current peaked, which means that ions were passing through the protein. After a few minutes, the signal decayed to its initial value. We interpret this observation as the signature of light-induced activation and subsequent deactivation of Arch-3. According to the literature, in any type of a rhodopsin photocycle, the deprotonation of the Schiff base after light excitation occurs within a range of picoseconds [38,39]. The deprotonation opens the channel; however, the subsequent recovery of the Schiff base requires milliseconds to several seconds [38,39,40,41]. Until recovery, the channel remains open for protons to pass through. Fitting an exponential decay to the curve from the time it begins to fall gives a recovery time τ of about 84 s.

**Figure 3:**
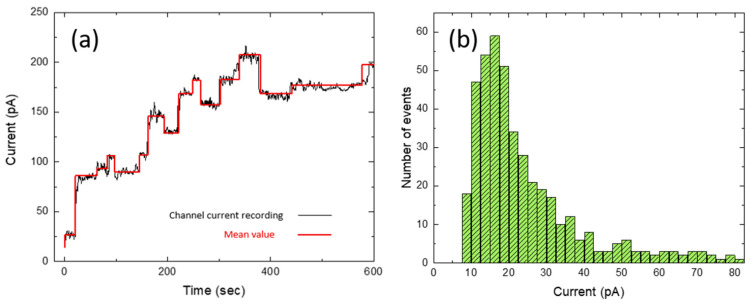
(**a**) Current signal as a function of time for an Arch-3-containing bilayer under continuous 532 nm laser illumination (black line). The red line was obtained by ‘’change point analysis’’ from the software Origin (Origin 2021b; OriginLab Corporation, Northampton, Massachusetts, USA). It shows the mean value of each current step. (**b**) The histogram shows the frequency of certain current steps grouped in intervals of 2.5 pA.

To characterize Arch-3 activation more deeply, we continuously exposed an Arch-3-containing bilayer to monochromatic light with a wavelength of 532 nm. During light exposure, we observed an overall increase in the conductance, which was composed of a stepwise increase and decrease in the current signal, as shown in Figure 3a. This signal is caused by a combination of opening and closing channels and the simultaneous fusion of Arch-3-EGFP containing vesicles from the aqueous phase with the bilayer. If Arch-3-EGFP is added, the current recording will expose a positive jump. The individual current steps ΔI, as indicated by the red line in Figure 3a, were obtained using the “change point analysis” algorithm as part of the software Origin (Origin 2021b; OriginLab Corporation, Northampton, MA, USA). In total, over 300 steps were analyzed. The histogram shown in Figure 3b displays the frequency of certain current steps ΔI grouped within intervals of 2.5 pA. From the histogram, it can be seen that most of the jumps were in the range of (16.25 ± 6.25) pA, where the full width at half maximum was used to determine the experimental uncertainty. The obtained value is in excellent agreement with the value found in the literature for bacteriorhodopsin (BR) [42]. The large current steps ΔI causing the asymmetric distribution towards larger current steps in the histogram could be due to multiple channels in the bilayer simultaneously being activated or deactivated.

Although Arch-3 is a proton pump, protons can passively pass while the channel is open. Considering the current step with the highest probability, i.e., the maximum peak of the histogram at Δ*I* = 16.25 pA, and assuming that Arch-3 has a passive opening in the bilayer, we can determine the radius of a single Arch-3 channel from the relation [43]

(1) $$r=\sqrt{\frac{l\Delta I}{\pi GCV}}$$

where l≈3 nm is the length of the protein assumed to be close to the thickness of the bilayer, ΔI = 16.25 pA is the amplitude of the typical current jump for one channel opening or closing, and V=20 mV is the applied voltage. The molarity C of the reaction solution was converted from the osmolarity, which was measured as 1.02 osmol/kg. Because of the complexity of the reaction solution, the number of dissociable particles per molecule (*n*) was assumed to be two, as is the case for NaCl. Thus, the molarity was obtained as C=0.5 M. Using this value, the molar conductivity results as G=18.4 S·M^−1^·m^−1^. The corresponding prediction of the pore radius of an Arch-3 channel, r≈(0.31 ±0.02) nm, is in excellent agreement with the radius of the pore of BR (0.4–1.1) nm [42] and of rhodopsin (0.45–0.7) nm [41], which also consist of seven transmembrane helices forming the same structure.

## 3. Conclusions

In this paper, we presented a simple and new microfluidic approach to investigate the electrophysiological properties of the recombinantly produced transmembrane protein Arch-3 inserted in a free-standing DOPC/DPhPC bilayer. By applying a voltage across such an Arch-3-containing DOPC/DPhPC bilayer, the light-induced opening of individual Arch-3 ion channels could be observed. The corresponding pore radius of the Arch-3 ion channel was determined to be (0.31 ± 0.02) nm, which is in excellent agreement with values found for similar protein pores.

The in vitro system described here presents the advantage of quick testing and prototyping of modified Arch-3, since only the DNA needs to be adapted. Moreover, even non-canonical amino acids can be incorporated [28,44,45]. Because G protein-coupled receptors involved in many signaling cascades exhibit a similar structure, we expect our work to be helpful for in vitro studies focusing on this kind of protein. This may pave the way for the creation of artificial signaling cascades.

## 4. Materials and Methods

### 4.1. Gene Expression

For gene expression, a commercially available cell-free expression system (*E. coli* T7 S30 Extract System for Circular DNA; Promega, Madison, Wisconsin, USA) was used. The plasmid in our experiments was VV020: WT Arch-3-EGFP in pET28b, a gift from Adam Cohen (Addgene plasmid # 58488; http://n2t.net/addgene:58488 (last accessed on 4 September 2021); RRID: Addgene_58488; Addgene, Watertown, Massachusetts, USA) [16]. The gene expression was performed in the presence of SUV (small unilamellar vesicles). For this, first a solution containing vesicles was prepared as follows: 400 µL of S30 Premix without Amino Acids, 50 µL of Amino Acid Mixture Minus Cysteine at 1 mM, 50 µL of Amino Acid Mixture Minus Leucine at 1 mM and 500 µL of ultrapure water were mixed. To this solution, 1 mg of DPhPC was added and sonicated three times, applying the continuous cycle of 4 s pulse, 4 s break for 4 min, and 2 min pause between each cycle. After that, the solution was put into the fridge to rest for 24 h.

Gene expression reactions were performed as follows: The components of the cell-free expression system were combined to obtain a “mastermix” containing 40 µL of S30 Premix without Amino Acids, 5 µL of Amino Acid Mixture Minus Cysteine at 1 mM, 5 µL of Amino Acid Mixture Minus Leucine at 1 mM and 30 µL of T7 S30 Extract System for Circular DNA. To this mastermix, 10 nM of plasmid DNA was added and filled up with vesicles solution to achieve a total reaction volume of 100 µL. The expression reaction was then incubated at 37 °C for 48 h. The synthesized Arch-3-EGFP was incorporated into the vesicles. Each reaction solution was directly injected into the microfluidic device after expression and could no longer be used after 24 h.

### 4.2. Lipid Preparation, and Device and Bilayer Fabrication

DOPC (1,2-dioleoyl-sn-glycero-3-phosphocholine), and DPhPC (1,2-diphytanoyl-sn-glycero-3-phosphocholine) were used for bilayer preparation. The lipids were from Avanti Polar Lipids (Avanti Polar Lipids, Birmingham, Alabama, USA). To prepare the oil–lipid solution, 5 mg of lipids (1:1 DOPC/DPhPC) were dissolved in 1 mL of pure squalene oil (Sigma-Aldrich, St. Louis, Missouri, USA) at 45 °C while undergoing continuous stirring for 3 h.

**Figure 4:**
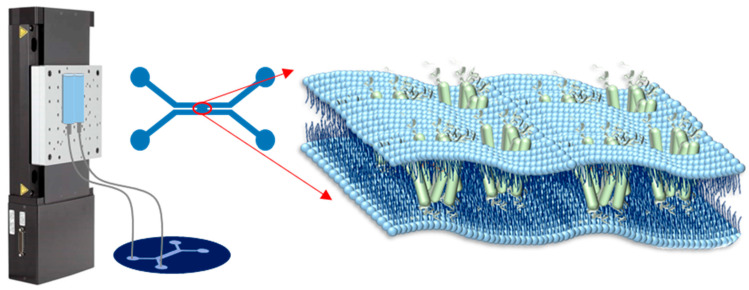
Design and structure of the microfluidic setup including the hydrostatic pressure system.

The microfluidic chip was produced by standard soft lithographic protocols and consisted of Sylgard 184 bonded to a glass slide, see e.g., ref. [24] for fabrication details. The chip was designed with two side-to-side rectangular channels with a width of 500 µm and height of 100 µm, forming an X geometry (see Figure 4).

The membrane was formed across an orifice with a width of about 150 µm that connected the two parallel channels, as sketched in Figure 4. A hydrostatic pressure system was used to control the flow of the aqueous solution in the microfluidic chip [24]. The two inlets were connected to two syringes, which were fixed on a motorized stage, and the two outlets were left open. By adjusting the height of the motorized stage, positive or negative pressures could be applied to the channels causing the aqueous solution to move forward or backward.

For bilayer formation, the whole chip was first filled with the squalene oil containing dissolved lipids. Subsequently, the cell-free expression reaction solution containing synthesized Arch-3-EGFP proteins that were fused to vesicles was injected gently into both microfluidic channels, displacing the oil but leaving behind an oil inclusion at the orifice connecting the microfluidic channels. During this process the two oil–water interfaces were being decorated with a monolayer of lipids and Arch-3-EGFP. Due to the drainage of oil into the PDMS, the two lipid monolayers came into contact with each other, leading to the formation of a bilayer. While bilayer formation, the Arch-3-EGFP at the interface of the two monolayers fuses into the bilayer (sketched in Figure 1).

### 4.3. Microscope Setup and Electrical Measurements

An inverted epifluorescence microscope (Axio Observer Z1; Zeiss, Oberkochen, Germany) with 473 nm (blue) and 532 nm (green) laser illumination was used. As Arch-3 was tagged with enhanced green fluorescent protein (EGFP), we used the wavelength of 473 nm to excite the EGFP and monitor Arch-3 production (see Figure 2b). The electrical properties of the Arch-3-EGFP-containing bilayer were analyzed by electrophysiological measurements using a patch-clamp amplifier, EPC 10 USB (Heka Electronics, Reutlingen, Germany). For that purpose, Ag/AgCl electrodes were prepared by inserting a 5 cm-long silver wire into a borosilicate glass pipet containing 150 mM of NaCl electrolyte solution while applying 5 V for 30 min. The prepared electrodes were inserted into the inlets of the microfluidic device. The current passing through the bilayer was measured over time using an excitation signal with an amplitude of 20 mV and a time resolution of 100 ms.
